# Colonial Medical Service

**Published:** 1916-09-02

**Authors:** 


### COLONIAL MEDICAL SERVICE.

Full details regarding this service and the advantages it offers to newly qualified men may be obtained on application to the Private Secretary, Colonial Office.

